# REM Sleep Behavior Disorder (RBD) in Dementia with Lewy Bodies (DLB)

**DOI:** 10.1155/2018/9421098

**Published:** 2018-06-19

**Authors:** Po-Chi Chan, Hsun-Hua Lee, Chien-Tai Hong, Chaur-Jong Hu, Dean Wu

**Affiliations:** ^1^Department of Neurology, Show Chwan Memorial Hospital, Changhua, Taiwan; ^2^Department of Neurology, Taipei Medical University Shuang Ho Hospital, New Taipei City, Taiwan; ^3^Department of Neurology, School of Medicine, College of Medicine, Taipei Medical University, Taipei, Taiwan; ^4^Sleep Center, Taipei Medical University Shuang Ho Hospital, New Taipei City, Taiwan; ^5^Vertigo and Balance Impairment Center, Taipei Medical University Shuang Ho Hospital, New Taipei City, Taiwan

## Abstract

Rapid eye movement sleep behavior disorder (RBD) is a parasomnia, with abnormal dream-enacting behavior during the rapid eye movement (REM) sleep. RBD is either idiopathic or secondary to other neurologic disorders and medications. Dementia with Lewy bodies (DLB) is the third most common cause of dementia, and the typical clinical presentation is rapidly progressive cognitive impairment. RBD is one of the core features of DLB and may occur either in advance or simultaneously with the onset of DLB. The association between RBD with DLB is widely studied. Evidences suggest that both DLB and RBD are possibly caused by the shared underlying synucleinopathy. This review article discusses history, clinical manifestations, possible pathophysiologies, and treatment of DLB and RBD and provides the latest updates.

## 1. Introduction

Lewy body dementia (LBD), including dementia with Lewy bodies (DLB) and Parkinson's disease dementia (PDD), is often discussed simultaneously because of the overlaps in pathological features, such as the presence of Lewy bodies and intracellular inclusions of *α*-synuclein and ubiquitin in the brain stem, limbic area, forebrain, and neocortex. It is still not clear whether DLB and PDD are distinct disorders or the same disease but at different stages. DLB is the third most common type of dementia in the world [1]. The annual incidence is 0.1% of the general population and 3.2% of all new dementia population [2]. The prevalence of DLB varied from 3.0% to 26.3% of patients who are above 65 years old [3, 4]. On the other hand, rapid eye movement sleep behavior disorder (RBD) is a disorder with abnormal behavior during the rapid eye movement (REM) sleep. RBD is acknowledged as a core clinical feature of DLB because the prevalence is up to 76% [5]. RBD not only precedes or coincides with the onset of DLB but also occurs during the course of the progression [6]. The cooccurrence of RBD, DLB, and Parkinson's disease is an important topic. In this review, we aimed to review the clinical features of DLB and its sleep manifestations. New advances in diagnosis, management, and underlying pathophysiology of DLB and RBD would also be discussed and summarized.

## 2. Dementia with Lewy Bodies

DLB was first clearly defined in 1996, at the First International Workshop of the Consortium on Dementia with Lewy Bodies [7]. The clinical diagnostic criteria include rapid progressive mental impairment to dementia as the central feature of DLB. Specific core features include fluctuated cognitive function, persistent well-formed visual hallucinations, and spontaneous motor features of parkinsonism. DLB is distinct from PDD, Alzheimer's disease (AD), and other types of dementia in several aspects, including clinical symptoms, pathological findings, and nuclear imaging studies [8]. The extrapyramidal symptoms of DLB are extremely sensitive to neuroleptic medications [7]. Lower dopamine transporter uptake in the basal ganglia is demonstrated by SPECT or PET imaging. They are also the suggestive features of DLB [9]. The confirmation of DLB is based on pathology which shows Lewy bodies in the brain stem or cerebral cortex [10]. Lewy-related neurites, Alzheimer pathology, and spongiform change may also be presented, but they are not necessary features for diagnosis.

In 2017, the diagnostic criteria have been updated for the fourth time (Table 1) [5]. The revised criteria had made some changes and defined essential diagnosis of dementia in a more detailed way. Dementia, defined by progressive cognitive decline of sufficient magnitude, interferes with normal social or occupational functions, or usual daily activities are still essential for the diagnosis of DLB. Unlike AD, patients are not necessary to have prominent or persistent memory impairment in the early stages, instead of dysfunction of attention, executive function, and visuoperceptual ability [11]. Core clinical features may precede dementia including fluctuating cognition with pronounced variations in attention and alertness, recurrent well-formed and detailed visual hallucinations, and RBD [12]. One or more spontaneous cardinal features of parkinsonism may also present such as bradykinesia, rest tremor, and rigidity. Supportive clinical features include severe sensitivity to antipsychotic agents, postural instability, repeated falls, syncope or other transient episodes of unresponsiveness, severe autonomic dysfunction, hyposmia, hallucinations in other modalities, delusions, apathy, anxiety, and depression and may exist in the early stage [13].

The revised DLB consensus criteria are now distinguished clearly between clinical features and diagnostic biomarkers. If one or more of the indicative biomarkers are identified and are associated with one or more core clinical features, then probable DLB should be diagnosed. Dementia without any core clinical features, but with one or more indicative biomarkers, may be classified as possible DLB. For the supportive biomarkers, these are biomarkers consistent with DLB that help the diagnostic evaluation, but without clear diagnostic specificity.

## 3. Recommended Management of DLB

With few randomized controlled trials in DLB, recommendations about clinical management are largely based upon expert opinions. Management of DLB is complex, and the approach with a multidisciplinary team is helpful. It is recommended that the combination of pharmacologic and nonpharmacologic approaches might result in an optimal outcome. Among others, exercise [14], cognitive training [15], and caregiver-oriented education and training are promising nonpharmacologic managements for DLB patients [16, 17]. For the pharmacologic therapy, there are four clinical aspects that should be considered:

1. Cognitive functions
2. It is well established that the involvement of cholinergic deficits in DLB and cholinesterase inhibitors (ChEIs) is beneficial for better clinical courses by lowering the choline degradation [18]. Either rivastigmine or donepezil may improve cognition, global function, and activities of living [19, 20]. On the other hand, there are evidences of hyperactivity of glutamatergic neurons in both human and animal studies [21, 22]. It is likely that memantine, the N-methyl-D-aspartate receptor antagonist, may be effective by amelioration of glutamatergic neurotransmission for cognitive improvement of DLB patients. However, memantine produced remarkable efficacy on global assessment, but the cognitive function, behavioral symptoms, and activities of daily living are based on current evidence [19].
3. Psychiatric symptoms
4. ChEIs may reduce apathy, visual hallucinations, delusion, and associated anxiety and agitation [23]. The use of antipsychotics should be avoided because of the increased risk of mortality [24]. The second generation of antipsychotics, such as quetiapine, may be beneficial and safer based on a previous study [25]. However, efficacy and tolerability are not well established yet. On the other hand, the incidence of depression ranged from 22 to 53 per 100 person-years in atypical PD, including DLB, which is higher as compared to PD [26]. Regarding the management of depression in DLB patients, serotoninergic-targeted therapy would be an option based on individual response. Furthermore, selective serotonin reuptake inhibitor treatment may even be associated with increased hippocampal neurogenesis and preservation of cognition in DLB/PDD patients according to a recent study [27].
5. Extrapyramidal symptoms
6. Previous studies mostly revealed no benefits or mild improvement with levodopa treatment in motor symptoms of DLB patients [28, 29]. The use of dopaminergic medications in DLB is often withheld by fears of aggravating psychosis and confusion. However, although the response to dopaminergic treatment in DLB for its motor symptoms is poor, a low dose of levodopa is still an alternative and should be titrated slowly in order to avoid psychiatric side effects [28, 30].
7. Sleep disturbances
8. DLB patients frequently have several sleep problems, and they often occur simultaneously. Excessive daytime sleepiness can be managed by improving sleep hygiene. Previous studies regarding central nervous system stimulants such as modafinil and armodafinil revealed inconsistent results [31]. Armodafinil has been associated with increased wakefulness but may exacerbate agitation and hallucinations [32]. It is critical to prevent sleep-related injuries in DLB patients with RBD. Taking clonazepam before bedtime may reduce the RBD and consequent injuries. However, it may worsen the cognitive function and daytime sleepiness.

## 4. REM Sleep Behavior Disorder (RBD)

The first major classification of sleep disorders, the Diagnostic Classification of Sleep and Arousal Disorders, was published in 1979 [33]. In 2014, the International Classification of Sleep Disorders (ICSD) made the 3rd revised version of the American Academy of Sleep Medicine's manual of sleep disorders nosology [34]. A parasomnia involves undesired events that happen during sleeping. It also involves three different classifications: nonrapid eye movement- (NREM-) related parasomnias, REM-related parasomnias, and other parasomnias. RBD is one of the REM-related parasomnias.

RBD was first described in 1986 by Schenck et al. [35]. In this 2-year study, 5 patients were found to have similar behavioral disturbances during REM sleep. They were associated with loss of chin and limb electromyography atonia [12, 36, 37]. RBD is either idiopathic or secondary [34]. Idiopathic RBD is cryptogenic since Lewy bodies were demonstrated by autopsy in two cases of presumptive idiopathic RBD [38, 39]. Idiopathic RBD is also found to have *α*-synuclein accumulation and thought to be the prodromal stage of many neurodegenerative disorders, such as PD and DLB, multiple system atrophy (MSA), and a pure autonomic failure [40]. Secondary RBD is defined to be caused by other diseases, most commonly in neurodegenerative or other neurologic disorders, sleep disorders, medications, and withdrawal states. Known causes of secondary RBD up to date are spinocerebellar ataxia [41], limbic encephalitis [42], brain tumors [42], multiple sclerosis [43], stroke [44], different antidepressants [45–47], alcohol [48], and barbiturate withdrawal [49].

## 5. Clinical Presentation of RBD

RBD has special clinical dream-enacting behavior symptoms such as repeated episodes of sleep-related vocalization and/or complex motor behaviors during REM sleep, correlating with dreams [50]. Patients with RBD act out their dreams and commonly have violent or injurious behavior during sleep [35, 51, 52]. The behavior includes purposeful movements of short duration and can be really violent thrashing, punching, kicking, and even falling out of the bed [53]. The patients also have loud vocalizations, screaming, and talking. Under normal conditions, the skeletal muscles lose muscle tone physiologically when dreaming [54]. In RBD, a loss of atonia causes self-injury and injury to their bed partners. Diagnosing RBD needs a careful and detailed history taking [55]. Males are much more than females in RBD patient groups. The median age at diagnosis is between 60 and 70 years [56]. The symptoms progress gradually; they can occur daily or once per year [57]. Dream contents of RBD patients are different from the normal population. Their dreams are mostly unpleasant and more violent. Patients do not usually consider this a problem, until themselves or their bed partners get hurt [58].

## 6. Clinical Implications of RBD and Its Relationship to DLB

RBD can be either idiopathic and a marker of prodromal neurodegeneration or secondary to neurodegeneration [59]. Longitudinal studies of idiopathic RBD had shown evidence of a strong association with eventual phenoconversion to a neurodegenerative disease. Phenoconversion risk between two and five years is about 15% to 35%, and the risk may increase to 41% to 90.9% if extending the follow-up period up to 12 to 25 years. When patients have RBD, compared to those without RBD, the accompanied neurodegenerative disease tends to be worse, such as more severe parkinsonian symptoms, autonomic dysfunction, and cognitive impairment [37].

Overall, idiopathic RBD patients convert to PD eventually and accomplish with 5 years. Those PD patients converted from RBD were faster motor progression, less response to levodopa therapy, and more severe postural instability [60, 61]. RBD appears to be associated with the *α*-synucleinopathies [62]. Previous studies showed that 38%–65% of RBD patients have developed *α*-synucleinopathy from 10 to 20 years after RBD presentation; it could be most likely PD, DLB, or multiple system atrophy [63, 64]. Other cohort studies have provided evidence that patients with idiopathic RBD will eventually develop a synucleinopathy, such as PD, DLB, or MSA [37, 40, 65–68].

Prevalence of RBD in the general population is between 0.38 and 0.5%, but RBD has been found in 70% of patients with MSA, 40% of patients with DLB, and between 15% and 33% of patients with PD [69–71]. Another study even showed that 74% of the DLB population had RBD [72, 73]. By using diffusion magnetic resonance imaging (MRI), PD patients with RBD showed microstructural white matter changes in the bilateral cingulum, inferior front occipital fasciculus, bilateral corticospinal tracts, and middle cerebellar peduncles. However, PD patients without RBD do not have such signal changes [74]. A series of autopsies of RBD patients shows underlying synucleinopathy up to 94% of patients [65, 75–77]. Furthermore, peripheral tissues such as submandibular glands and the enteric nervous system from living patients with idiopathic RBD also show abnormal *α*-synuclein immunoreactivity [78, 79]. The implication from these evidences aforementioned is that RBD onset in older adults is associated with underlying synucleinopathy in pathology.

## 7. Diagnoses and Treatment of RBD

The diagnosis of RBD is based on clinical features and polysomnography (PSG) findings. Firstly, the patients must have repeated episodes of complex motor behaviors or vocalization during REM sleep, and they can be documented by PSG or reports of dream enactment. Secondly, it must have evidence of REM sleep without atonia on PSG, namely, REM sleep without atonia (RSWA). Other clinical findings are strongly suggested by the diagnosis of RBD, but RSWA is not observed, yet the diagnosis can still be given. RBD can be secondary to several medications, most of which are antidepressants [80, 81]. When RBD is believed as a secondary cause of medication use, a diagnosis of RBD is still applicable.

The management of RBD should be applied carefully because around 33% to 65% RBD patients have sleep-related injuries that are caused by the patients themselves [82]. Sleep disruption usually happens because of abnormal or disruptive, or even violent, behavior during REM sleep [83]. So, modifying the sleep environment is necessary for RBD patients to prevent from getting sleep-related injuries. Under certain circumstances, separating bedrooms may be necessary to prevent bed partners from getting injuries. Pharmacologic therapy includes clonazepam and melatonin [82, 84]. Both are modestly effective in preventing sleep-related injuries. Clonazepam is a long-acting benzodiazepine with increased risks of falls and cognitive impairment. It should be used with caution in RBD patients with dementia, gait disorders, or concomitant obstructive sleep apnea. Melatonin is a hormone releasing from the pineal gland with favorable safety and tolerability. Thus, RBD patients not suitable for clonazepam should consider melatonin as their first-line treatment [85]. Other dopaminergic medications, such as pramipexole, paroxetine, or levodopa, show no therapeutic effect, sometimes even worsen RBD [86]. Only few studies have discussed about the drug effect of ChEIs [87]. They are considered to treat RBD patients with a concomitant synucleinopathy. Other medications are used for RBD treatment, with less evidence, such as zopiclone, benzodiazepines, desipramine, clozapine, carbamazepine, and sodium oxybate [88–91].

## 8. Pathophysiology of RBD

Sleep is a physiological state, which is defined by consciousness loss, reduced responsiveness to the environment, and decreased body movement [66]. Two different states with different biochemical, neuronal, and metabolic properties are involved in sleep: non-REM sleep and REM sleep. REM sleep was named because of its most obvious behavior—rapid eye movements during sleep [92, 93]. Besides rapid eye movement, one of the features of REM is muscle atonia, which means the muscle activity is inhibited, except middle ear muscle activity and eye movement. A recent study has proved that the modulation of dorsal medulla GABAergic neurons control the REM phenomenon in rodents [94]. The atonia is believed to be controlled by nuclei in the lower pons and medulla, particularly the perilocus ceruleus area. This area directly innervates the spinal interneurons and induces REM atonia via a flip-flop switch [32]. During REM sleep, excitatory sublateral dorsal/subcoeruleus nucleus glutamatergic neurons activate spinal cord inhibitory interneurons to hyperpolarize and thereby inhibit the spinal motoneuron pool and cause REM sleep atonia. It is proposed that hypocretin neurons, located in the posterior hypothalamus, may further stabilize the REM-active and REM-inactive centers [95].

The key structure for modulating REM sleep is the brain stem, particularly the pons and adjacent portions of the midbrain. During REM sleep, cholinergic neurons in the pontine tegmentum of the reticular formation are active. GABAergic neurons and orexin/hypocretin neurons also regulate REM sleep [96]. REM sleep has special EEG characteristics with low-amplitude desynchronized theta activity with the presence of saw-tooth waves, the presence of pontine–geniculate–occipital (PGO) waves, and hippocampal theta activity. PGO waves occur during the transition from non-REM sleep to REM sleep or during REM sleep itself. REM sleep is the sleep state with the highest physiological arousal.

Currently, the loss of REM sleep atonia control is well accepted in RBD patients. However, the underlying mechanisms for RBD or RSWA are far from clear. Motor circuits involve afferent, integrative, and efferent systems in the spinal cord, the brain stem, the basal ganglia, the cerebellum, and the limbic/cerebral cortex. Abnormal activation and disinhibition of these motor circuits may explain this sleep-related motor manifestation. During REM sleep, there are physiologic intermittent muscle twitches that could be originated from the red nucleus, pedunculopontine nucleus, and lateral dorsal tegmental nucleus. Dysfunction of sublateral dorsal/subcoeruleus nucleus from brain lesions in the dorsal pons was proved to cause RSWA and may cause clinical RBD [97, 98]. It is proposed that the accumulation of Lewy bodies in the pontine and medullary structures, which is in advance to the degeneration of the substantia nigra according to the Braak stage [99], will result in RSWA and RBD. With the ascending of Lewy bodies to the substantia nigra and cortex, the motor symptoms and dementia evolved [100]. On the other hand, regarding DLB, before the development of parkinsonism and other features, RBD typically begins many years before cognitive dysfunction. It can be explained that the nigrostriatal system is later or less involved in DLB as compared to PD and PDD, and the neocortex is involved in an earlier phase.

Animal experiments can help understand the biological mechanism, pathogenesis, and treatment of RBD. In 1965, Jouvet and Delorme first reported a cat model of loss of REM atonia, which was induced by bilateral, symmetrical, and mediodorsal pontine tegmental electrolytic injury [101]. These 35 pontine-impaired cats have de novo “hallucinatory-type” behavior during REM sleep. During non-REM sleep, the hallmark phasic REM sleep PGO waves were frequently seen [102]. Jouvet's group then discovered that bilateral lesions of the caudal and ventral pons were descending pathways for REM atonia [103]. Morrison's group designed an idiopathic RBD cat and dog animal model and discovered clonazepam as an effective therapeutic medication [35, 104]. Valenica Garcia et al. developed the RBD rat model for pharmacotherapies of clonazepam and melatonin [82]. Also, the rat model can be used for studying other medication effects, such as antidepressants [105]. Currently, Lyon's group developed a novel rat model of RBD with precision techniques that deepen the understanding of the human RBD basic and clinical research [106].

The specific neuronal networks and neuropathology involved in human RBD pathogenesis have not been identified with certainty; more work is needed for determination. RBD shares similar pathological changes with PD and DLB in sleep-related brainstem nuclei, specifically those in the rostral pontine tegmentum. In PD and DLB, *α*-synuclein immunoreactive Lewy-related pathology and neuronal loss are discovered in the same area [107–109]. But DLB with or without RBD differs clinically or pathologically. DLB without RBD has early involvement of corticolimbic areas and later extension to brainstem nuclei. DLB with RBD has early brainstem involvement then extends to forebrain structures [110–112]. AD-type neuropathology is frequent in DLB. Some of the DLB and PD patients, especially those with PD with later developing dementia, can have *β*-amyloid plaques and tau-positive neurofibrillary degeneration, which belongs to AD. RBD is uncommon in AD, but when RBD occurred, *α*-synuclein deposition is discovered [113]. Another study showed that probable RBD is associated with DLB and less severe AD-related pathology in the medial temporal lobes. If the absence of probable RBD, AD-like atrophy patterns on MRI and increased phospho-tau burden are noted [114, 115]. The cholinergic mechanism is an important modulatory role of RBD. Cholinergic depletion is also known to be severe in DLB, even in the early beginning of the dementia [116]. Loss of neurons in the locus coeruleus and substantia nigra in DLB with subsequent dysregulation of cholinergic neurons in the pedunculopontine nucleus increased REM sleep drive and RBD [116, 117]. The pedunculopontine/laterodorsal tegmental nuclei (PPN/LDT) are active during REM sleep which contain cholinergic neurons. In an animal study, damage in PPN/LDT leads to RBD-like behavior. Cholinergic neurons decrease, and *α*-synuclein accumulation in the PPN/LDT is seen in DLB with and without RBD, also in PD and AD. But different from AD, significant neuronal loss was only detected in these nuclei in DLB [69, 77, 118–120]. With the help of these innovative research methodologies and materials, further insight of mechanisms of pathogenesis can be expected in the near future.

## 9. Conclusions

Compelling evidence has suggested that both DLB and RBD are possibly caused by underlying synucleinopathy, and RBD often precedes DLB. Currently, the mainstay of pharmacological treatment of RBD is clonazepam and melatonin, which may reduce the possibility of sleep-related injuries. As a sentinel of possible DLB and other synucleinopathy, RBD may be the beginning of degenerative diseases and provide opportunities for preemptive treatment for earlier disease-modifying therapy, targeting *α*-synuclein or its regulatory pathways. Further studies need to address factors that will determine its phenoconversion to different types of synucleinopathy. Early interventional strategies should be developed to prevent or at least delay the consequences of devastating dementia, parkinsonism, and autonomic failures.

## Figures and Tables

**Table 1:** Revised [7, 121] criteria for the clinical diagnosis of probable and possible dementia with Lewy bodies (DLB).

*Essential* for a diagnosis of DLB is dementia, defined as a progressive cognitive decline of sufficient magnitude to interfere with normal social or occupational functions, or with usual daily activities. Prominent or persistent memory impairment may not necessarily occur in the early stages but is usually evident with progression. Deficits on tests of attention, executive function, and visuoperceptual ability may be especially prominent and occur early.

*Core clinical features (The first 3 typically occur early and may persist throughout the course.)*
(i) Fluctuating cognition with pronounced variations in attention and alertness (ii) Recurrent visual hallucinations that are typically well formed and detailed (iii) REM sleep behavior disorder, which may precede cognitive decline (iv) One or more spontaneous cardinal features of parkinsonism: these are bradykinesia (defined as slowness of movement and decrement in amplitude or speed), rest tremor, or rigidity

*Supportive clinical features*
Severe sensitivity to antipsychotic agents; postural instability; repeated falls; syncope or other transient episodes of unresponsiveness; severe autonomic dysfunction, for example, constipation, orthostatic hypotension, urinary incontinence; hypersomnia; hyposmia; hallucinations in other modalities; systematized delusions; apathy, anxiety, and depression

*Indicative biomarkers*
(i) Reduced dopamine transporter uptake in basal ganglia demonstrated by SPECT or PET (ii) Abnormal (low uptake) ^123^iodine-MIBG myocardial scintigraphy (iii) Polysomnographic confirmation of REM sleep without atonia

*Supportive biomarkers*
(i) Relative preservation of medial temporal lobe structures on CT/MRI scan (ii) Generalized low uptake on SPECT/PET perfusion/metabolism scan with reduced occipital activity 6 the cingulate island sign on FDG-PET imaging (iii) Prominent posterior slow-wave activity on EEG with periodic fluctuations in the prealpha/theta range

*Probable DLB* can be diagnosed if
(a) Two or more core clinical features of DLB are present, with or without the presence of indicative biomarkers, or
(b) Only one core clinical feature is present, but with one or more indicative biomarkers.

*Probable DLB should not be diagnosed on the basis of biomarkers alone.*
*Possible DLB* can be diagnosed if
(a) Only one core clinical feature of DLB is present, with no indicative biomarker evidence, or
(b) One or more indicative biomarkers is present but there are no core clinical features.

*DLB* is less likely
(a) In the presence of any other physical illness or brain disorder including cerebrovascular disease sufficient to account in part or in total for the clinical picture, although these do not exclude a DLB diagnosis and may serve to indicate mixed or multiple pathologies contributing to the clinical presentation, or
(b) If parkinsonian features are the only core clinical feature and appear for the first time at a stage of severe dementia.

DLB should be diagnosed when dementia occurs before or concurrently with parkinsonism. The term Parkinson disease dementia (PDD) should be used to describe dementia that occurs in the context of well-established Parkinson disease. In a practice setting, the term that is most appropriate to the clinical situation should be used and generic terms such as Lewy body disease are often helpful. In research studies in which distinction needs to be made between DLB and PDD, the existing 1-year rule between the onset of dementia and parkinsonism continues to be recommended.

(Adapted from Ian G. McKeith et al., Neurology 89 July 4, 2017).
